# $\text{Sech}^{2}$-type solitary waves and the stability analysis for the KdV–mKdV equation

**DOI:** 10.1038/s41598-024-67317-x

**Published:** 2024-07-15

**Authors:** Zhi-Guo Liu, Muhua Liu, Jinliang Zhang

**Affiliations:** 1 School of Mathematics and Statistics, Henan University of Science and Technology, Luoyang, 471000 China; 2 Postdoctoral Innovation Practice Base, Henan CAERI Vehicle Testing and Certification Center Co., Ltd, Jiaozuo, 454000 China

**Keywords:** Applied mathematics, Fluid dynamics

## Abstract

In this article, we investigated the solitary wave solutions of the KdV–mKdV equation using Hirota’s bilinear method. Closed-form analytical single and multiple solitary wave solutions were obtained. Through qualitative methods and the analysis of solitary waveforms, we discovered that in addition to sech-type solitary waves, the system also contains $\text{Sech}^{2}$-type solitary waves. By employing the trial functions method, we obtained a single $\text{Sech}^{2}$-type solitary wave and verified its existence and stability using the split-Step Fourier Transform method. Furthermore, we use the collision of two $\text{Sech}^{2}$-type single solitary waves to excite a stable $\text{Sech}^{2}$-type double solitary wave. Similarly, we excite a stable triple solitary wave with three $\text{Sech}^{2}$-type single solitary waves. This method can also be used to excite stable multiple solitary waves. It is shown that these solitary wave solutions enrich the dynamic behavior of the KdV–mKdV equation and provide methods for solving $\text{Sech}^{2}$-type solitary waves, which hold significant theoretical value.

## Introduction

The study of solutions for nonlinear partial differential equations (NLPDEs) is essential to the theory of physical mathematics and theoretical physics^1^. These solutions help us to better understand the mechanisms of complicated nonlinear physical phenomena and the dynamic processes modeled by these nonlinear evolution equations^2^. The KdV equation (Korteweg-de Vries equation) and the mKdV equation (modified Korteweg-de Vries equation) are two important types of nonlinear partial differential equations, widely applied in many physical systems. In some cases, these two equations can be combined to better describe complex physical phenomena. The combined KdV–mKdV equation is an important tool for describing nonlinear wave phenomena, with widespread applications in many fields, such as solid-state physics, plasma physics, fluid physics and quantum field theory^3–5^. By studying these equations, we can gain a deeper understanding and predict the nonlinear behavior in complex systems, thereby advancing related science and technology. The combined equations not only enrich the theoretical foundation of nonlinear science but also provide powerful tools for solving practical problems. These combined equations play a significant role in the study of nonlinear waves, solitons, and other complex systems.

The existence of soliton-type solutions for nonlinear models is highly significant due to their potential applications in various physics areas, including nonlinear optics, plasmas, fluid mechanics, condensed matter, and many more. In fact, many kinds of soliton solutions have been obtained by using for example, the inverse scattering method^6^, the Sub-ODE method^7^, the tanh method^8^, the Darboux transformation^9^, the Lie group method^10^, the algebrogeometric method^11^, the homogeneous balance method^12^ and the Hirota’s method^13^, The extended rational expansion method^14^, The MSE method^15^ and so on.

The combined form of the Korteweg-de Vries equation and the Modified Korteweg-de Vries equation form the following so-called combined KdV–mKdV equation^3,1^:

1 $$\begin{aligned} {u_t} + \alpha u{u_x} + \beta {u^2}{u_x} + \gamma {u_{xxx}} = 0, \end{aligned}$$

where $\alpha$, $\beta$ and $\gamma$ are constants. Specifically, $\alpha$ controls the strength of the first-order nonlinear effects in the equation, $\beta$ adjusts the strength of the second-order nonlinear effects, typically describing stronger nonlinear effects, $\gamma$ controls the strength of the dispersion effects of waves.

The KdV–mKdV equation is completely integrable, giving rise to multiple-soliton solutions. The combined KdV–mKdV equation and its generalizations have attracted increasing interest, as evidenced by numerous studies^16–22^. The authors obtained tanh–sech type solitary solutions and triangular functions solutions in KdV–mKdV equation in Ref.^23^. In Ref.^24^, the tanh, coth and tan type solitary wave solutions were studied. In Ref.^5^, the periodic progressive wave, and one and two soliton solutions were studied by various analytical methods. In Ref.^25^, the bell-shaped soliton and a new soliton solution were obtained by a kinds of Jacobi doubly periodic solutions. Moreover, new periodic wave solutions were constructed by a method with the aid of a Sub-ODE. The rational solutions with free multi-parameters and N-soliton solutions were obtained by the bilinear approach in Ref.^26^. Although nonlinear solitary waves in the KdV–mKdV equations have been studied extensively both analytically and numerically, there are still many open problems. For instance, we have well-developed methods for solving and analyzing soliton waves of the sech type. However, to our knowledge, there is no research on $\text{Sech}^{2}$-type solitary waves, including the existence and stability of $\text{Sech}^{2}$-type solitary waves in the KdV–mKdV equations.

In this work, we consider the generalized combined KdV–mKdV equation (1). By employing the method of trial functions, we derived several approximate analytic solutions of $\text{Sech}^{2}$-type solitary waves. Through the application of perturbation methods^27,28^, we investigated the stability of both single and multiple $\text{Sech}^{2}$-type solitary waves, and successfully obtained stable solitary wave solutions for both single and multiple $\text{Sech}^{2}$-types.

## The bilinear form

The Hirota’s bilinear method is an effective approach for obtaining analytical solutions to nonlinear evolution equations. Therefore, we used Hirota’s bilinear method to solve Eq. (1).

In order to find the soliton-like solutions of Eq. (1) we set

2 $$\begin{aligned} u = {a_0} + i{\left( {\ln \frac{{{f^*}}}{f}} \right) _x}, \end{aligned}$$

where ${a_0}$ is a constant to be determined later, and ${i^2} = - 1$ , $f = a + bi,{f^*} = a - bi$ , $a,b \in R$ .

Substituting the Eq. (2) into Eq. (1) yields

3 $$\begin{aligned} \begin{array}{l} {\left( {\ln \frac{{{f^*}}}{f}} \right) _{xt}} + (\alpha {a_0} + \beta a_0^2){\left( {\ln \frac{{{f^*}}}{f}} \right) _{xx}} + i(\alpha + 2\beta {a_0}){\left( {\ln \frac{{{f^*}}}{f}} \right) _x}{\left( {\ln \frac{{{f^*}}}{f}} \right) _{xx}} - \beta {\left[ {{{\left( {\ln \frac{{{f^*}}}{f}} \right) }_x}} \right] ^2}{\left( {\ln \frac{{{f^*}}}{f}} \right) _{xx}} + \gamma {\left( {\ln \frac{{{f^*}}}{f}} \right) _{xxxx}} = 0{.} \end{array} \end{aligned}$$

Integrating both sides of Eq. (3) by x once and setting the constant of integration equal to zero yields:

4 $$\begin{aligned} {\left( {\ln \frac{{{f^*}}}{f}} \right) _t} + \gamma {\left( {\ln \frac{{{f^*}}}{f}} \right) _{xxx}} + (\alpha {a_0} + \beta a_0^2){\left( {\ln \frac{{{f^*}}}{f}} \right) _x} + \frac{1}{2}i(\alpha + 2\beta {a_0}){\left[ {{{\left( {\ln \frac{{{f^*}}}{f}} \right) }_x}} \right] ^2} - \frac{1}{3}\beta {\left[ {{{\left( {\ln \frac{{{f^*}}}{f}} \right) }_x}} \right] ^3} = 0{.} \end{aligned}$$

Expanding the Eq. (4) leads to

5 $$\begin{aligned} \begin{array}{l} \frac{{f_t^*f - {f^*}{f_t}}}{{f{f^*}}} + \gamma \left[ {\frac{{f_{xxx}^*}}{{{f^*}}} - \frac{{{f_{xxx}}}}{f} + 2{{\left( {\frac{{f_x^*}}{{{f^*}}}} \right) }^3} - 2{{\left( {\frac{{{f_x}}}{f}} \right) }^3} + 3\frac{{{f_x}{f_{xx}}}}{{{f^2}}} - 3\frac{{f_x^*f_{xx}^*}}{{{{\left( {{f^*}} \right) }^2}}}} \right] \\ \quad +\left( {\alpha {a_0} + \beta a_0^2} \right) \frac{{f_x^*f - {f^*}{f_x}}}{{f{f^*}}} + \frac{1}{2}i\left( {\alpha + 2\beta {a_0}} \right) {\left( {\frac{{f_x^*f - {f^*}{f_x}}}{{f{f^*}}}} \right) ^2} - \frac{\beta }{3}{\left( {\frac{{f_x^*f - {f^*}{f_x}}}{{f{f^*}}}} \right) ^3} = 0. \end{array} \end{aligned}$$

By a simple transformation, Eq. (5) can be transformed into the following equation:

6 $$\begin{aligned} \begin{array}{l} \frac{{f_t^*f - {f^*}{f_t}}}{{f{f^*}}} + \gamma \left[ {\frac{{ff_{xxx}^* - {f^*}{f_{xxxx}} - 3f_{xx}^*{f_x} + 3f_x^*{f_{xx}}}}{{{f^*}f}}} \right] + \gamma \left[ {\frac{{ - 3f_{xx}^*{f_x} + 3f_x^*{f_{xx}}}}{{{f^*}f}}} \right] + \gamma \left[ {3\frac{{{f_x}{f_{xx}}}}{{{f^2}}} - 3\frac{{f_x^*f_{xx}^*}}{{{{\left( {{f^*}} \right) }^2}}}} \right] \\ \quad + \gamma \left[ {2{{\left( {\frac{{f_x^*}}{{{f^*}}}} \right) }^3} - 2{{\left( {\frac{{{f_x}}}{f}} \right) }^3}} \right] + \left( {\alpha {a_0} + \beta a_0^2} \right) \frac{{f_x^*f - {f^*}{f_x}}}{{f{f^*}}} + \frac{1}{2}i\left( {\alpha + 2\beta {a_0}} \right) {\left( {\frac{{f_x^*f - {f^*}{f_x}}}{{f{f^*}}}} \right) ^2} - \frac{\beta }{3}{\left( {\frac{{f_x^*f - {f^*}{f_x}}}{{f{f^*}}}} \right) ^3} = 0. \end{array} \end{aligned}$$

By substitution Eq. (6) into Eq. (1) and using the properties of the bilinear operator, we obtain

7 $$\begin{aligned} \begin{array}{l} \left[ {{D_t} + \gamma D_x^3 + \left( {\alpha {a_0} + \beta a_0^2} \right) {D_x}} \right] ({f^*} \cdot f) + \gamma (2 - \frac{\beta }{3})({f^*}f)\left[ {{{\left( {\frac{{f_x^*}}{{{f^*}}}} \right) }^3} - {{\left( {\frac{{{f_x}}}{f}} \right) }^3}} \right] \\ \quad - \frac{3}{{{f^*}f}}{D_x}({f^*} \cdot f)\left[ {{{\left( {f_{xx}^*f - \frac{\beta }{3}f_x^*{f_x} + {f^*}f} \right) }_{xx}} - \frac{1}{6}i\left( {\alpha + 2\beta {a_0}} \right) {D_x}({f^*} \cdot f)} \right] = 0. \end{array} \end{aligned}$$

In order to obtain the bilinear form of Eq. (7), setting $\beta = 6$ , then we have

8 $$\begin{aligned} \left[ {{D_t} + \gamma D_x^3 + \left( {\alpha {a_0} + 6a_0^2} \right) {D_x}} \right] ({f^*} \cdot f) - \frac{3}{{{f^*}f}}{D_x}({f^*} \cdot f)\left\{ {\left[ {D_x^2 - \frac{1}{6}i\left( {\alpha + 12{a_0}} \right) {D_x}} \right] ({f^*} \cdot f)} \right\} = 0. \end{aligned}$$

Further, we obtain the bilinear form of Eq. (8):

9 $$\begin{aligned} \left\{ \begin{array}{l} \left[ {{D_t} + \gamma D_x^3 + \left( {\alpha {a_0} + 6a_0^2} \right) {D_x}} \right] ({f^*} \cdot f) = 0,\\ {D_x}({f^*} \cdot f)\left\{ {\left[ {D_x^2 - \frac{1}{6}i\left( {\alpha + 12{a_0}} \right) {D_x}} \right] ({f^*} \cdot f)} \right\} = 0. \end{array} \right. \end{aligned}$$

It should be noted that the bilinear form Eq. (9) is obtained when the coefficient $\beta = 6$.

According to the Hirota’s bilinear method , we may choose the *f* and ${f^*}$ in the form

10 $$\begin{aligned} \left\{ \begin{array}{l} f = 1 + \varepsilon {f^{(1)}} + {\varepsilon ^2}{f^{(2)}} + \cdots + {\varepsilon ^n}{f^{(n)}} + \cdots , \\ {f^ * } = 1 + \varepsilon {f^{(1) * }} + {\varepsilon ^2}{f^{(2) * }} + \cdots + {\varepsilon ^n}{f^{(n) * }} + \cdots . \end{array} \right. \end{aligned}$$

Substituting Eq. (10) into Eq. (9) and collecting coefficients of polynomials of with the aid of *Mathematica*, then equating the coefficients of various powers of $\varepsilon$ to zero, we get a set of algebraic equations:

11 $$\begin{aligned}{} & {} \epsilon ^1: {\left\{ \begin{array}{ll} \gamma (f^{(1)^*}-f^{(1)})_{xxx}+(\alpha a_0+6a_0^2)(f^{(1)^*}-f^{(1)})_x+(f^{(1)^*}-f^{(1)})_t=0,\\ (f^{(1)^*}+f^{(1)})_{xx}-\frac{1}{6}i(\alpha +12a_0)(f^{(1)^*}-f^{(1)})_t=0, \end{array}\right. } \end{aligned}$$

12 $$\begin{aligned}{} & {} \epsilon ^2: {\left\{ \begin{array}{ll} \gamma (f^{(2)^*}-f^{(2)})_{xxx}+(\alpha a_0+6a_0^2)(f^{(2)^*}-f^{(2)})_x+(f^{(2)^*}-f^{(2)})_t=-[D_t+(\alpha a_0+6a_0^2)D_x+D_x^3]f^{(1)^*}f^{(1)},\\ (f^{(2)^*}+f^{(2)})_{xx}-\frac{1}{6}i(\alpha +12a_0)(f^{(2)^*}-f^{(2)})_x= -[D_x^2-\frac{1}{6}i(\alpha +12a_0)D_x+\gamma D_x^3]f^{(1)^*}f^{(1)}. \end{array}\right. } \end{aligned}$$

13 $$\begin{aligned}{} & {} \epsilon ^3: {\left\{ \begin{array}{ll} \gamma (f^{(3)^*}-f^{(3)})_{xxx}+(\alpha a_0+6a_0^2)(f^{(3)^*}-f^{(3)})_x+(f^{(3)^*}-f^{(3)})_t=\\ -[D_t+(\alpha a_0+6a_0^2)D_x+\gamma D_x^3](f^{(1)^*}f^{(2)}+f^{(2)^*}f^{(1)}),\\ (f^{(3)^*}+f^{(3)})_{xx}-\frac{1}{6}i(\alpha +12a_0)(f^{(3)^*}-f^{(3)})_x=\\ -[D_x^2-\frac{1}{6}i(\alpha +12a_0)D_x+\gamma D_x^3](f^{(1)^*}f^{(2)}+f^{(2)^*}f^{(1)}). \end{array}\right. } \end{aligned}$$

Suppose that

14 $$\begin{aligned} {f^{(1)}} = {\mathrm{{e}}^{{\xi _1} + i{\theta _1}}}, \end{aligned}$$

where ${\xi _1} = {\omega _1}t + {k_1}_`x + {c_1}$, ${\theta _1}$ are constants to be determined later

Substituting Eq. (14) into Eq. (11), we have

15 $$\begin{aligned} {\omega _1}&= - \gamma k_1^3 - (\alpha {a_0} + 6a_0^2){k_1}{,} \end{aligned}$$

16 $$\begin{aligned} 6k_1^2\cos {\theta _1}&= (\alpha + 12{a_0})\sin {\theta _1}. \end{aligned}$$

## Analytical solution

Based on the bilinear form of the KdV–mKdV equation, we can obtain analytical solutions for single and double solitary waves.

### Single solitary wave solutions

For single soliton solutions we set ${f^{(2)}} = {f^{(3)}} = \cdots = {f^{(n)}} = \cdots = 0$ , ${f^{(1)}} = 1 + {\mathrm{{e}}^{{\xi _1} + i{\theta _1}}}$ and $\varepsilon = 1$ , then we have

17 $$\begin{aligned} \left\{ \begin{array}{l} f = 1 + {f^{(1)}}, \\ {f^ * } = 1 + {f^{(1) * }}. \end{array} \right. \end{aligned}$$

Substituting Eq. (17) into Eq. (2), the single soliton solutions are given by

18 $$\begin{aligned} {u_1} = {a_0} + i{\left( {\ln \frac{{{f^*}}}{f}} \right) _x} = {a_0} + i{\left( {\ln \frac{{1 + {\mathrm{{e}}^{{\xi _1} - i{\theta _1}}}}}{{1 + {\mathrm{{e}}^{{\xi _1} + i{\theta _1}}}}}} \right) _x}, \end{aligned}$$

which can be rewritten as

19 $$\begin{aligned} {u_1} = {a_0} + \frac{{2{k_1}{\mathrm{{e}}^{{\xi _1}}}\sin {\theta _1}}}{{1 + {\mathrm{{e}}^{2{\xi _1}}} + 2{\mathrm{{e}}^{{\xi _1}}}\cos {\theta _1}}}, \end{aligned}$$

where

20 $$\begin{aligned} {\omega _1} = - \gamma k_1^3 - (\alpha {a_0} + 6a_0^2){k_1},\ k_1^2\cos {\theta _1} = \frac{1}{6}(\alpha + 12{a_0})\sin {\theta _1}. \end{aligned}$$

### Double solitary wave solutions

Suppose that

21 $$\begin{aligned} {f^{(1)}} = {\mathrm{{e}}^{{\xi _1} + i{\theta _1}}} + {\mathrm{{e}}^{{\xi _2} + i{\theta _2}}}, \end{aligned}$$

where ${\xi _i} = {\omega _i}t + {k_i}_`x + {c_i}$ , $${\theta _i}, \begin{array}{*{20}{c}} {i \in \{ 1,2\} } \end{array}$$ are constants to be determined later.

Substituting Eq. (21) into Eq. (12), we have

22 $$\begin{aligned} {f^{(2)}} = {\mathrm{{e}}^{{\xi _1} + {\xi _2} + i{\theta _1} + i{\theta _2} + {A_{12}}}},{\mathrm{{e}}^{{A_{12}}}} = {\left( {\frac{{{k_1} - {k_2}}}{{{k_1} + {k_2}}}} \right) ^2}. \end{aligned}$$

For double soliton solutions we set ${f^{(3)}} = {f^{(4)}} = \cdots = {f^{(n)}} = \cdots = 0$ and $\varepsilon = 1$ , then we have

23 $$\begin{aligned} \left\{ \begin{array}{l} f = 1 + {\mathrm{{e}}^{{\xi _1} + i{\theta _1}}} + {\mathrm{{e}}^{{\xi _2} + i{\theta _2}}} + {\mathrm{{e}}^{{\xi _1} + {\xi _2} + i{\theta _1} + i{\theta _2} + {A_{12}}}},\\ {f^ * } = 1 + {\mathrm{{e}}^{{\xi _1} - i{\theta _1}}} + {\mathrm{{e}}^{{\xi _2} - i{\theta _2}}} + {\mathrm{{e}}^{{\xi _1} + {\xi _2} - i{\theta _1} - i{\theta _2} + {A_{12}}}}. \end{array} \right. \end{aligned}$$

Substituting Eq. (23) into Eq. (2), we obtain the double solitary wave solutions:

24 $$\begin{aligned} {u_2} = {a_0} + i{\left( {\ln \frac{{{f^*}}}{f}} \right) _x} = {a_0} + i{\left( {\ln \frac{{1 + {\mathrm{{e}}^{{\xi _1} - i{\theta _1}}} + {\mathrm{{e}}^{{\xi _2} - i{\theta _2}}} + {\mathrm{{e}}^{{\xi _1} + {\xi _2} - i{\theta _1} - i{\theta _2} + {A_{12}}}}}}{{1 + {\mathrm{{e}}^{{\xi _1} + i{\theta _1}}} + {\mathrm{{e}}^{{\xi _2} + i{\theta _2}}} + {\mathrm{{e}}^{{\xi _1} + {\xi _2} + i{\theta _1} + i{\theta _2} + {A_{12}}}}}}} \right) _x}, \end{aligned}$$

where ${\omega _i} = - \gamma k_i^3 - (\alpha {a_0} + 6a_0^2){k_i}$, $k_i^2\cos {\theta _i} = \frac{1}{6}(\alpha + 12{a_0})\sin {\theta _i}$, $${\theta _i}, \begin{array}{*{20}{c}} {i \in \{ 1,2\} }, \end{array}$$ are constants to be determined later.

## $\text{Sech}^{2}$-type solitary wave solutions

Using $0 \le \left| {\cos \theta } \right| \le 1$ from Eq. (19), we have

25 $$\begin{aligned} {a_0} + \frac{{2{k_1}{\mathrm{{e}}^{{\xi _1}}}\sin {\theta _1}}}{{1 + {\mathrm{{e}}^{2{\xi _1}}} + 2{\mathrm{{e}}^{{\xi _1}}}}} \le {a_0} + \frac{{2{k_1}{\mathrm{{e}}^{{\xi _1}}}\sin {\theta _1}}}{{1 + {\mathrm{{e}}^{2{\xi _1}}} + 2{\mathrm{{e}}^{{\xi _1}}}\cos {\theta _1}}} \le {a_0} + \frac{{2{k_1}{\mathrm{{e}}^{{\xi _1}}}\sin \theta _1 }}{{1 + {\mathrm{{e}}^{2{\xi _1}}}}}, \end{aligned}$$

and it can be transformed into

26 $$\begin{aligned} {a_0} + {k_1}\sin {\theta _1}{{\mathop {\text{ s ech}}\nolimits } ^2}({\xi _1}/2) \le {a_0} + \frac{{2{k_1}{\mathrm{{e}}^{{\xi _1}}}\sin {\theta _1}}}{{1 + {\mathrm{{e}}^{2{\xi _1}}} + 2{\mathrm{{e}}^{{\xi _1}}}\cos {\theta _1}}} \le {a_0} + {k_1}\sin {\theta _1}{\mathop {\text{ s ech}}\nolimits } {\xi _1}. \end{aligned}$$

From the above qualitative analysis, it is easy to conclude that the waveform of solution (19) lies between that of sech-type and $\text{Sech}^{2}$-type solitary waves.

When $\sin {\theta _1} = 1$, solution (19) reduces to the standard sech-type solitary wave solutions

27 $$\begin{aligned} {u_1} = {a_0} + \frac{{2{k_1}{\mathrm{{e}}^{{\xi _1}}}}}{{1 + {\mathrm{{e}}^{2{\xi _1}}}}} = {a_0} + {k_1}\frac{2}{{{\mathrm{{e}}^{ - {\xi _1}}} + {\mathrm{{e}}^{{\xi _1}}}}} = {a_0} + {k_1}{\mathop {\text{ s ech}}\nolimits } {\xi _1}. \end{aligned}$$

In the limit case when ${\theta _1} \rightarrow 0$ ($\sin {\theta _1} \sim {\theta _1}$, $\cos {\theta _1} \approx 1$ ) the solution (1) become the $\text{Sech}^{2}$-type solitary wave solutions

28 $$\begin{aligned} {u_1} = {a_0} + \frac{{2{k_1}{\theta _1}}}{{{{\left( {1 + {\mathrm{{e}}^{{\xi _1}/2}}} \right) }^2}}} = {a_0} + {k_1}{\theta _1}{{\mathop {\text{ s ech}}\nolimits } ^2}({\xi _1}/2). \end{aligned}$$

It is worth noting that due to ${\theta _1}$ possibly approaching zero, the solution may have significant errors. Solution (28) represents a specific analytical solution. To obtain a more general $\text{Sech}^{2}$-type solitary wave solution, we need to adjust the parameters of solution (19) and solve again.

Suppose that

29 $$\begin{aligned} {u_1} = u({\xi _1}) = {a_0} + \frac{{2{k_1}{\mathrm{{e}}^{{\xi _1}}}M\sin {\theta _1}}}{{1 + {\mathrm{{e}}^{2{\xi _1}}} + 2{\mathrm{{e}}^{{\xi _1}}}\cos {\theta _1}}}, \end{aligned}$$

which can be rewritten as

30 $$\begin{aligned} {u_1} = u({\xi _1}) = {a_0} + \frac{{2{k_1}M\sin {\theta _1}}}{{{\mathrm{{e}}^{ - {\xi _1}}} + {\mathrm{{e}}^{{\xi _1}}} + 2\cos {\theta _1}}} = {a_0} + \frac{{{k_1}M\sin {\theta _1}}}{{1/({\mathop {\text{ s ech}}\nolimits } {\xi _1}) + \cos {\theta _1}}} = {a_0} + {G^{ - 1}}, \end{aligned}$$

where $$ \begin{array}{{c}} {{\xi _1} = {k_1}x - {\omega _1}}t, \end{array} + {c_1}$$, $G = \frac{{1/({\mathop {\text{ s ech}}\nolimits } {\xi _1}) + \cos {\theta _1}}}{{{k_1}M\sin {\theta _1}}}$, ${a_0}$, *a* and *M* are constants to be determined.

Substituting Eq. (30) into Eq. (1), we obtain

31 $$\begin{aligned}&- \omega k({a_0} + {G^{ - 1}}) + \frac{\alpha }{2}k(a_0^2 + 2{a_0}{G^{ - 1}} + {G^{ - 2}}) + \frac{\beta }{3}k(a_0^3 + 3a_0^3{G^{ - 1}} + 3{a_0}{G^{ - 2}} + {G^{ - 3}})\nonumber \\&+ \gamma {k^3}(2{G^{ - 3}}G_{{\xi _1}}^2 - {G^{ - 2}}{G_{{\xi _1}{\xi _1}}}) = 0. \end{aligned}$$

Further simplification of Eq. (31) yields:

32 $$\begin{aligned}&\frac{8}{{{{{\mathop {\text{ s ech}}\nolimits } }^3}{\xi _1}}}\left[ {\frac{1}{{{{(2{k_1}M\sin {\theta _1})}^3}}}( - {\omega _1}{a_0} + \frac{\alpha }{2}a_0^2 + \frac{\beta }{3}a_0^3)} \right] \nonumber \\&\quad + \frac{4}{{{{{\mathop {\text{ s ech}}\nolimits } }^2}{\xi _1}}}\left[ {\frac{{3{a_1}}}{{{{(2{k_1}M\sin {\theta _1})}^3}}}( - {\omega _1}{a_0} + \frac{\alpha }{2}a_0^2 + \frac{\beta }{3}a_0^3) + \frac{1}{{{{(2{k_1}M\sin {\theta _1})}^2}}}( - {\omega _1} + \alpha {a_0} + \beta a_0^2) + \frac{{\gamma k_1^2}}{{{{(2{k_1}M\sin {\theta _1})}^2}}}} \right] \nonumber \\&\quad + \frac{2}{{{\mathop {\text{ s ech}}\nolimits } {\xi _1}}}\left[ \frac{{3{{(2\cos {\theta _1})}^2}}}{{{{(2{k_1}M\sin {\theta _1})}^3}}}( - {\omega _1}{a_0} + \frac{\alpha }{2}a_0^2 + \frac{\beta }{3}a_0^3) + \frac{{2(2\cos \theta )}}{{{{(2{k_1}M\sin {\theta _1})}^2}}}( - {\omega _1} + \alpha {a_0} + \beta a_0^2)\right. \nonumber \\&\left. \quad + \frac{(\frac{\alpha }{2} + \beta {a_0})}{{2{k_1}M\sin {\theta _1}}} + \frac{{ - (2\cos \theta )\gamma k_1^2}}{{{{(2{k_1}M\sin {\theta _1})}^2}}} \right] \nonumber \\&\quad + \left[ \frac{{{{(2\cos {\theta _1})}^3}}}{{{{(2{k_1}M\sin {\theta _1})}^3}}}( - {\omega _1}{a_0} + \frac{\alpha }{2}a_0^2 + \frac{\beta }{3}a_0^3) + \frac{{{{(2\cos \theta )}^2}}}{{{{(2{k_1}M\sin {\theta _1})}^2}}}( - {\omega _1} + \alpha {a_0} + \beta a_0^2) \right. \nonumber \\&\left. \quad + \frac{{2\cos \theta (\frac{\alpha }{2} + \beta {a_0})}}{{2{k_1}M\sin {\theta _1}}} + \frac{{ - 8\gamma k_1^2}}{{{{(2{k_1}M\sin {\theta _1})}^2}}} + \frac{\beta }{3} \right] = 0. \end{aligned}$$

Collecting all terms with the powers in ${{\mathop {\text{ s ech}}\nolimits } ^{ - n}}\xi (n = 0,1,2,3)$ and setting each of the obtained coeffificients for ${{\mathop {\text{ s ech}}\nolimits } ^{ - n}}\xi$ to zero yields the following set of algebraic equations with respect to ${a_0}, {k_1}, {\omega _1}, \alpha , \beta , {\theta _1}$ and *M* :

33 $$\begin{aligned}&\frac{1}{{{{(2{k_1}M\sin {\theta _1})}^3}}}( - {\omega _1}{a_0} + \frac{\alpha }{2}a_0^2 + \frac{\beta }{3}a_0^3) = 0, \end{aligned}$$

34 $$\begin{aligned}&\frac{{3(2\cos {\theta _1})}}{{{{(2{k_1}M\sin {\theta _1})}^2}}}( - {\omega _1}{a_0} + \frac{\alpha }{2}a_0^2 + \frac{\beta }{3}a_0^3) + \frac{1}{{{{(2{k_1}M\sin {\theta _1})}^2}}}( - {\omega _1} + \alpha {a_0} + \beta a_0^2) + \frac{{\gamma k_1^2}}{{{{(2{k_1}M\sin {\theta _1})}^2}}} = 0, \end{aligned}$$

35 $$\begin{aligned}&\frac{{3{{(2\cos {\theta _1})}^2}}}{{{{(2{k_1}M\sin {\theta _1})}^3}}}( - {\omega _1}{a_0} + \frac{\alpha }{2}a_0^2 + \frac{\beta }{3}a_0^3) + \frac{{2(2\cos \theta )}}{{{{(2{k_1}M\sin {\theta _1})}^2}}}( - {\omega _1} + \alpha {a_0} + \beta a_0^2) \nonumber \\&\quad + \frac{1}{{2{k_1}M\sin {\theta _1}}}(\frac{\alpha }{2} + \beta {a_0}) + \frac{{ - (2\cos \theta )\gamma k_1^2}}{{{{(2{k_1}M\sin {\theta _1})}^2}}} = 0, \end{aligned}$$

36 $$\begin{aligned}&\frac{{{{(2\cos {\theta _1})}^3}}}{{{{(2{k_1}M\sin {\theta _1})}^3}}}( - {\omega _1}{a_0} + \frac{\alpha }{2}a_0^2 + \frac{\beta }{3}a_0^3) + \frac{{{{(2\cos \theta )}^2}}}{{{{(2{k_1}M\sin {\theta _1})}^2}}}( - {\omega _1} + \alpha {a_0} + \beta a_0^2) \nonumber \\&\quad + \frac{{2\cos \theta }}{{2{k_1}M\sin {\theta _1}}}(\frac{\alpha }{2} + \beta {a_0}) + \frac{{ - 8\gamma k_1^2}}{{{{(2{k_1}M\sin {\theta _1})}^2}}} + \frac{\beta }{3} = 0. \end{aligned}$$

Solving Eqs. (33)–(36), we obtain the following solutions:

Case (i):

37 $$\begin{aligned} {a_0} = 0, {\omega _1} = \gamma k_1^2, \frac{{{\alpha ^2}}}{{18\gamma {k^2}}} - \frac{{2\gamma }}{{{M^2}{{\sin }^2}{\theta _1}}} = - \frac{\beta }{3}. \end{aligned}$$

The travelling wave solution of KdV–MKdV equation are given by

38 $$\begin{aligned} {u_1} = {k_1}M\frac{{\sin {\theta _1}}}{{1/({\mathop {\text{ s ech}}\nolimits } {\xi _1}) + \cos {\theta _1}}}. \end{aligned}$$

(a) When $${\theta _1} = \pi /2$$, then

39 $$\begin{aligned} {u_1} = \pm \mathrm{{M}}{k_1}{\mathop {\text{ s ech}}\nolimits } {\xi _1}, \end{aligned}$$

where $${\omega _1} = \gamma k_1^2,\,{\xi _1} = {k_1}x - {\omega _1}t + {c_1}$$.

(b) When $${\theta _1} \rightarrow 0$$, then

40 $$\begin{aligned} {u_1} = \frac{{2{k_1}M\sin {\theta _1}}}{{{\mathrm{{e}}^{ - {\xi _1}}} + {\mathrm{{e}}^{{\xi _1}}} + 2\cos {\theta _1}}} \approx \frac{{2{k_1}M{\theta _1}}}{{{\mathrm{{e}}^{ - {\xi _1}}} + {\mathrm{{e}}^{{\xi _1}}} + 2}} = \frac{{{k_1}M{\theta _1}}}{{2/({{{\mathop {\text{ s ech}}\nolimits } }^2}\frac{{{\xi _1}}}{2})}} = \frac{1}{2}{k_1}M{\theta _1}{{\mathop {\text{ s ech}}\nolimits } ^2}\frac{{{\xi _1}}}{2}, \end{aligned}$$

where $${\omega _1} = \gamma k_1^2,\,{\xi _1} = {k_1}x - {\omega _1}t + {c_1}$$.

Case (ii): $\omega = - \gamma k_1^2 - \frac{1}{3}\beta a_0^2,\frac{\alpha }{2} + \beta {a_0} = 3\gamma k_1^2\frac{{\cos {\theta _1}}}{{{k_1}M\sin {\theta _1}}}, \gamma \left( {\frac{{2{{\cos }^2}{\theta _1}}}{{{M^2}{{\sin }^2}{\theta _1}}} - \frac{2}{{{M^2}{{\sin }^2}\theta }}} \right) + \frac{\beta }{3} = 0$, then

41 $$\begin{aligned} {u_1} = {a_0} + \frac{{{k_1}M\sin {\theta _1}}}{{1/({\mathop {\text{ s ech}}\nolimits } {\xi _1}) + \cos {\theta _1}}}, \end{aligned}$$

where $$\omega = - \gamma k_1^2 - \frac{1}{3}\beta a_0^2,\,{\xi _1} = {k_1}x - {\omega _1}t + {c_1}$$.

(a) When ${\theta _1} = \pi /2$, then ${a_0} = - \frac{\alpha }{{2\beta }},M = \pm \sqrt{\frac{{6\gamma }}{\beta }}$, we obtain

42 $$\begin{aligned} {u_1} = - \frac{\alpha }{{2\beta }} \pm \sqrt{\frac{{6\gamma }}{\beta }} {k_1}{\mathop {\text{ s ech}}\nolimits } {\xi _1}, \end{aligned}$$

where

43 $$\begin{aligned} \omega = - \gamma k_1^2 - \frac{1}{3}\beta {a_0},\,{\xi _1} = {k_1}x - {\omega _1}t + {c_1}. \end{aligned}$$

(b) When $${\theta _1} \rightarrow 0$$, then

44 $$\begin{aligned} {u_1} \approx {a_0} + \frac{{2{k_1}{\mathrm{{e}}^{{\xi _1}}}M{\theta _1}}}{{1 + {\mathrm{{e}}^{2{\xi _1}}} + 2{\mathrm{{e}}^{{\xi _1}}}\cos {\theta _1}}} = {a_0} + {k_1}M{\theta _1}{{\mathop {\text{ s ech}}\nolimits } ^2}{\xi _1}/2, \end{aligned}$$

where

45 $$\begin{aligned} \omega = - \gamma k_1^2 - \frac{1}{3}\beta {a_0},\,{\xi _1} = {k_1}x - {\omega _1}t + {c_1}. \end{aligned}$$

## The linear stability of solitary wave solutions of $\text{Sech}^{2}$-type

The Split-Step Fourier Transform (SSFT) method is a pseudospectral numerical technique utilized for solving nonlinear partial differential equations. This method involves computing the solution in incremental steps. It handles dispersive and nonlinear effects independently through a sequential approach: first addressing the nonlinear effects and then the dispersive effects.

In this section, we will use the split-step Fourier transform (SSFT) method^27^ to study the linear stability of single and multiple $\text{Sech}^{2}$-type solitary waves in equation (1). To this end, we use the waveform of a $\text{Sech}^{2}$-type solitary wave (refer to solution (40) or (44)) at time *t*=0 (selecting waveforms at other times is also acceptable and does not affect the analysis results) with a perturbation of a random uniformly distributed noise field of amplitude $10^{-4}$ as the initial condition. By observing the changes in perturbations over time, we can determine the stability of the solitary wave. If the perturbations are suppressed, the solitary wave is stable; if the perturbations increase exponentially, the solitary wave is unstable^27–30^.

Solution (28) is obtained under ${\theta _1} \rightarrow 0$ , but a key issue is that the wave number $k_1$ must be very large to ensure the validity of the solution. Therefore, we solved Equation (1) again and obtained Solution (40) or (44), which ensures a reasonable value for $k_1$.

Based on the above reasons, it is reasonable to use $\text{Sech}^{2}$-type solitary waves as the input signal without considering the dispersion relationship to study the stability of solitary waves.

**Figure 1:**
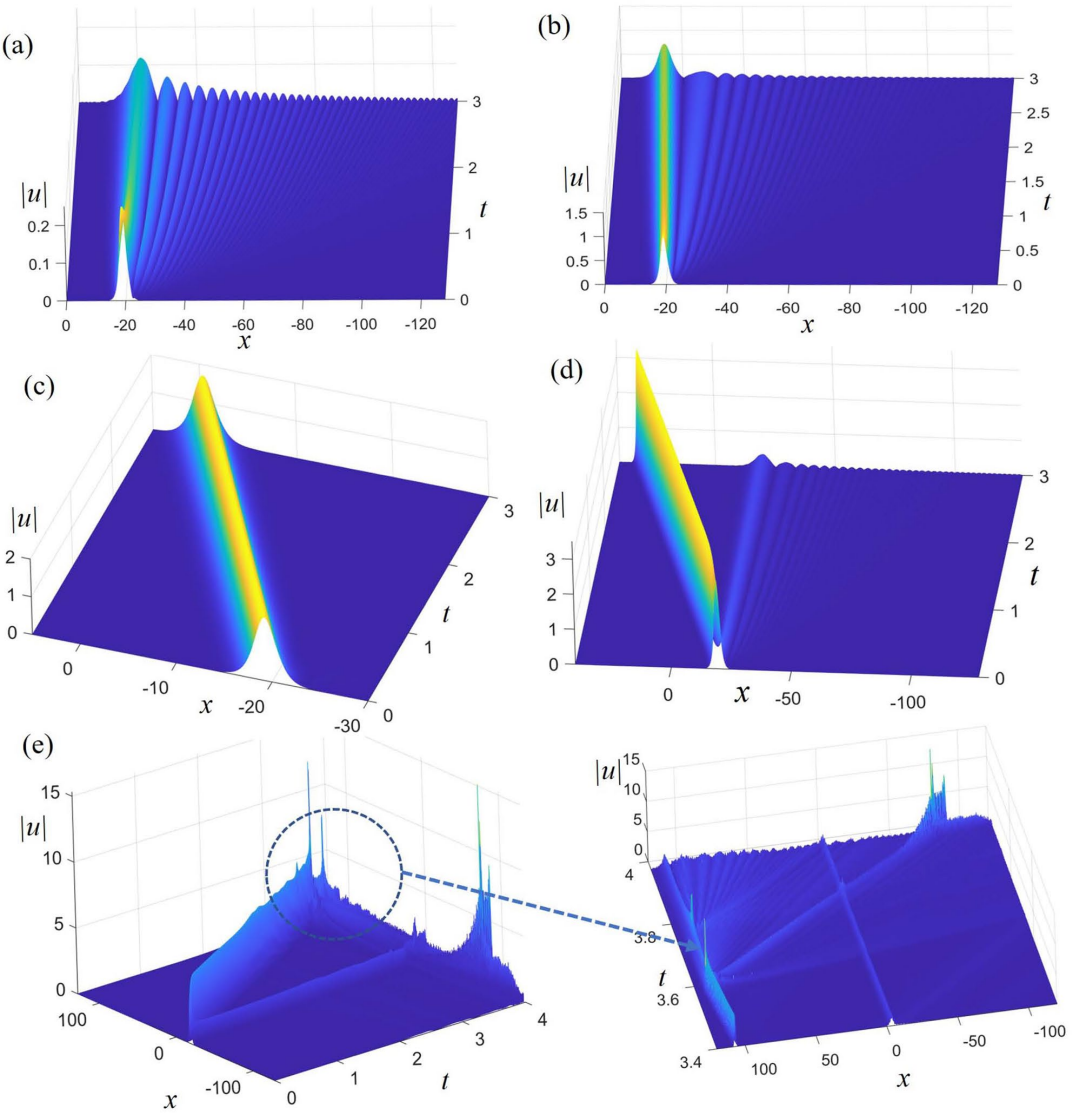
Stability analysis of a $\text{Sech}^{2}$-type solitary wave inspired by $u_D = D{{\mathop {\text{ s ech}}\nolimits } ^2}\left( {(1.3x - 10.7367t + 5)/2} \right)$ in Eq. (1) with $\alpha = - 6,\beta = 6, \gamma = 4.887$ , where (**a–e**) correspond to the amplitude *D* of − 0.3380, − 1.014, − 1, − 2.535, and − 3.4983 respectively.

Figure 1 illustrates the stability analysis of the $\text{Sech}^{2}$-type solitary wave $u_D = D{{\mathop {\text{ s ech}}\nolimits } ^2}\left( {(1.3x - 10.7367t + 5)/2} \right)$ (selected based on the solitary wave solution (40)) using SSFT method in Eq. (1) with $\alpha = - 6,\,\beta = 6\, \mathrm{{and \,}} \gamma = 4.887$. We assume an initial excitation which is the displacement field from $u_D$ at time *t* = 0 with a uniformly distributed random perturbation of amplitude $10^{-4}$^27,28^. The parameter D gradually increased with values of − 0.3380, − 1.014, − 1, − 2.535, and − 3.4983.

Figure 1a shows a stable train of solitary waves excited by a $\text{Sech}^{2}$-type solitary wave as the initial condition, with nearly thirty solitary waves from left to right, gradually decreasing in amplitude. From the perspective of energy conservation, the waveform of the solitary wave with the maximum amplitude (on the far left) no longer maintains a $\text{Sech}^{2}$ shape. Therefore, although we used a $\text{Sech}^{2}$-type solitary wave as the initial condition, stable $\text{Sech}^{2}$-type solitary waves were not excited.

Relative to Fig. 1a, the solitary waves in the stable train shown in Fig. 1b not only exhibit reduced amplitude variations but also decrease in number. The amplitude of the excited solitary wave (on the far left, with the maximum amplitude) in Fig. 1b more closely resembles a $\text{Sech}^{2}$ waveform.

The stable solitary wave excited in Fig. 1c does not show any changes compared to the initial waveform, clearly indicating the excitation of a $\text{Sech}^{2}$-type solitary wave.

Relative to Fig. 1a,b, the stable solitary wave excited in Fig. 1d (on the far left) has a larger amplitude, and the train of solitary waves in Fig. 1d contains fewer solitary waves with smaller amplitudes. It is evident that the solitary wave excited in Fig. 1d does not exhibit a standard $\text{Sech}^{2}$ shape. However, it is a stable solitary wave.

In Fig. 1e, we can see the initial solitary wave splitting into two solitary waves. Before $t=3.5$, both of these solitary waves maintain stable propagation. However, at $t=3.5$, the solitary wave on the left suddenly increases in amplitude and changes direction. It is evident from the figure that stable solitary waves were not excited.

In Eq. (1), the amplitude of the solitary wave is a factor that affects the stability as shown in Fig. 1. It can be observed that for the same solitary wave, as the wave amplitude increases gradually, the solitary wave becomes stable initially. However, with further continuous increase in wave amplitude, the solitary wave begins to become unstable.

We use the conclusion from Ref.^29^, “the double-soliton is inspired by the elastic collision of two single-solitons”, to construct a $\text{Sech}^{2}$-type double solitary wave.

To study the stability of $\text{Sech}^{2}$-type double solitary waves, we examine the collision of the following two single-soliton waves with different amplitudes: $u_{D1} = - 1.21{{\mathop {\text{ s ech}}\nolimits } ^2}\left( {0.5(x - 4.887t -10)} \right)$ and $u_{D2} = - 1.69{{\mathop {\text{ s ech}}\nolimits } ^2}\left( {0.5(1.3x - 10.7367t+5)} \right)$ at time $t = -2$, subjected to a uniformly distributed random perturbation of amplitude $10^{-4}$. The simulation results are presented in Fig. 1a.

In the same way, we use three $\text{Sech}^{2}$-type solitary waves $u_{D1}$ , $u_{D2}$ and $u_{D3} = - 1.8{{\mathop {\text{ s ech}}\nolimits } ^2}\left( {0.5(1.3x - 10.7367t+5)+10} \right)$to generate triple solitary waves, use four $\text{Sech}^{2}$-type solitary waves $u_{D1}$, $u_{D2}$, $u_{D3}$ and $u_{D4} = - 2.1{{\mathop {\text{ s ech}}\nolimits } ^2}\left( {0.5(1.3x - 10.7367t+35)} \right)$ to generate quadruple solitary waves, and use five $\text{Sech}^{2}$-type solitary waves $u_{D1}$, $u_{D2}$, $u_{D3}$, $u_{D4}$ and $u_{D5} = - 1.21{{\mathop {\text{ s ech}}\nolimits } ^2}\left( {0.5(x - 4.887t -20)} \right)$ to generate quintuple solitary waves.

**Figure 2:**
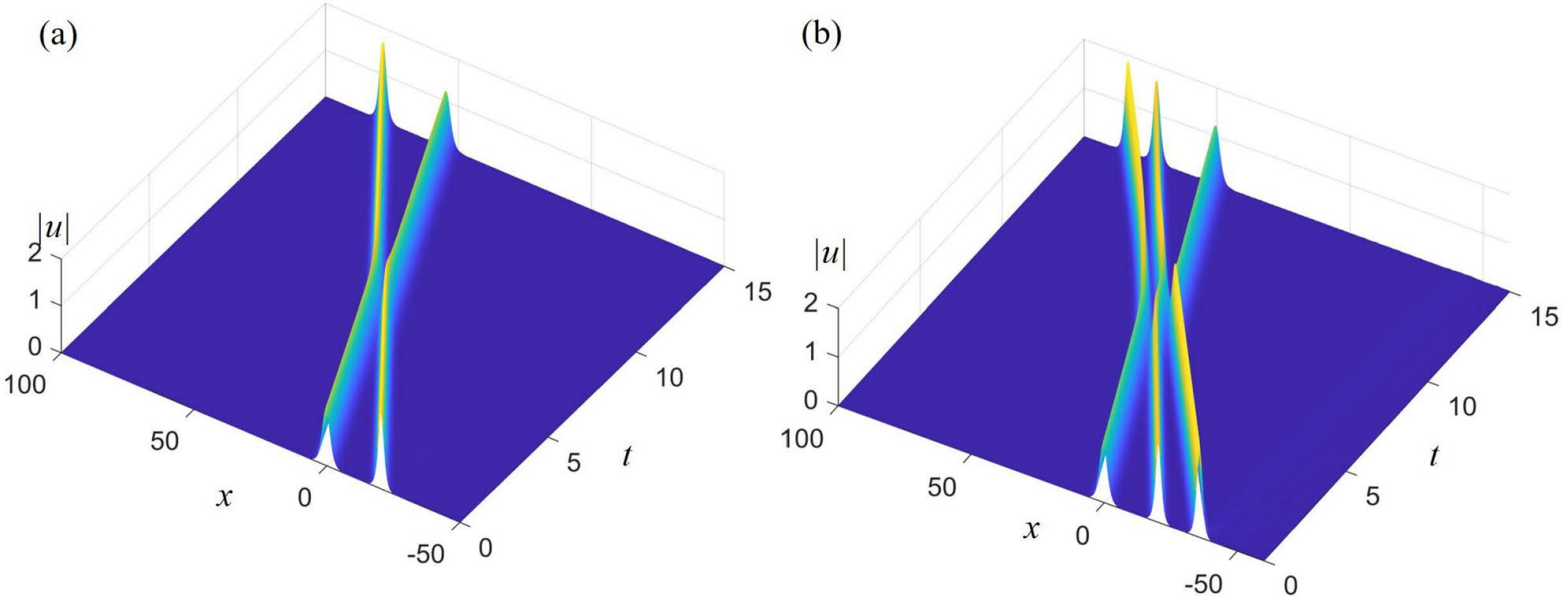
Stability analysis of $\text{Sech}^{2}$-type double-soliton or triple-soliton inspired by a perturbed excitation with $\alpha = - 6,\beta = 6, \gamma = 4.887$ : (**a**) initial condition selected from two single-soliton solutions $u_{D_1}$ and $u_{D_2}$; (**b**) initial condition selected from three single-soliton solutions $u_{D_1}$, $u_{D_2}$ and $u_{D_3}$.

**Figure 3:**
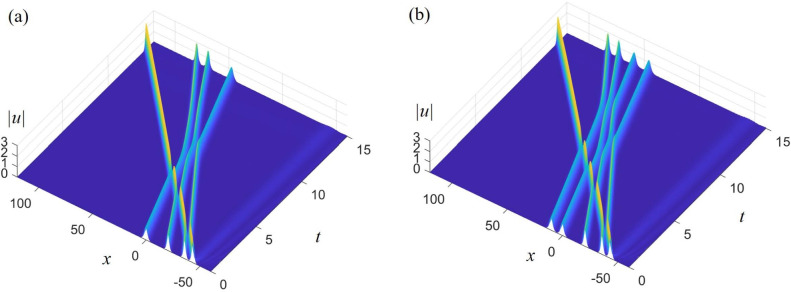
Stability analysis of $\text{Sech}^{2}$-type quadruple-soliton or quintuple-soliton inspired by a perturbed excitation with $\alpha = - 6,\beta = 6, \gamma = 4.887$ : (**a**) initial condition selected from four single-soliton solutions $u_{D_1}$, $u_{D_2}$, $u_{D_3}$ and $u_{D_4}$ ; (**b**) initial condition selected from five single-soliton solutions $u_{D_1}$, $u_{D_2}$, $u_{D_3}$, $u_{D_4}$ and $u_{D_5}$.

Figure 2a shows stable $\text{Sech}^{2}$-type double-solitary waves excited by the interaction of the initial single solitary waves $u_{D_1}$ and $u_{D_2}$. It is evident that the amplitudes of $u_{D_1}$ and $u_{D_2}$ do not change, indicating that the excited double solitary waves are of the $\text{Sech}^{2}$ type. Figure 2b utilizes the same method as Fig. 2a, using the interaction of single solitary waves $u_{D_1}$, $u_{D_2}$, and $u_{D_3}$ to excite triple solitary waves. It is easy to see from the figure that stable triple solitary waves of the $\text{Sech}^{2}$-type are excited. Figure 3 shows the stable quadruple solitary and quintuple solitary waves excited using the same method as in Fig. 2. Of course, if the initial conditions are chosen improperly, unstable multiple solitary waves may also be excited.

## Conclusion

This paper investigates the solitary wave solutions of the nonlinear KdV–MKdV equation using the Hirota’s bilinear method, focusing on both single solitary wave solutions and multiple solitary wave solutions. We know that the nonlinear terms of the KdV and mKdV equations often coexist in certain physical systems, such as fluid dynamics and quantum field theory, giving rise to the KdV–mKdV equation. Interestingly, in the general KdV equation, exact solitary wave solutions of the $\text{Sech}^{2}$-type exist, whereas in the mKdV equation, exact solitary wave solutions of the sech-type are present. The KdV–mKdV equation supports exact solitary wave solutions of the sech-type but does not support general exact solitary wave solutions of the $\text{Sech}^{2}$-type. Therefore, investigating the existence and stability of solitary waves of the $\text{Sech}^{2}$-type, both single and multiple, is of significant importance.

In the limit of analytic solutions, the $\text{Sech}^{2}$-type solitary waves can be obtained. However, this limitation requires a very large wave number. To obtain general $\text{Sech}^{2}$-type solitary waves, this paper re-solves the KdV–mKdV equation using the method of trial functions, resulting in general $\text{Sech}^{2}$-type solitary waves. Additionally, the propagation stability of single and multiple $\text{Sech}^{2}$-type solitary waves is analyzed and simulated using the SSFT, leading to stable single and multiple solitary waves.

This paper analyzes the impact of solitary wave amplitude on stability, finding that smaller amplitudes result in a train of solitary waves. As the amplitude increases, this train of solitary waves gradually disappears. Interestingly, when the amplitude reaches a certain level, $\text{Sech}^{2}$-type solitary waves are produced. Further increasing the amplitude causes perturbations to be exponentially amplified, which disrupts the solitary wave shape and leads to instability. Although we found stable $\text{Sech}^{2}$-type single and multiple solitary waves, our study revealed that the unstable solitary wave scenarios are very complex and may lead to rogue waves, bifurcations and chaos^31–34^. This will be the focus of our further research.

## Data Availability

The datasets used and/or analyzed during the current study are available from the corresponding author on reasonable request.
